# Testing the Chemical/Structural Stability of Proton Conducting Perovskite Ceramic Membranes by *in Situ*/*ex Situ* Autoclave Raman Microscopy 

**DOI:** 10.3390/membranes3040311

**Published:** 2013-10-25

**Authors:** Aneta Slodczyk, Oumaya Zaafrani, Matthew D. Sharp, John A. Kilner, Bogdan Dabrowski, Olivier Lacroix, Philippe Colomban

**Affiliations:** 1 Laboratory of Dynamics, Interactions and Reactivity (LADIR), UMR7075 CNRS, Université Pierre et Marie Curie, 4 Pl. Jussieu, Paris 75005, France; E-Mails: aneta.slodczyk@upmc.fr (A.S.); missoum83@yahoo.fr (O.Z.); 2 Department of Materials, Imperial College London, London SW7 2AZ, UK; E-Mails: m.sharp09@imperial.ac.uk (M.D.S.); j.kilner@imperial.ac.uk (J.A.K.); 3 Department of Physics, Northern Illinois University, DeKalb, IL 60115, USA; E-Mail: dabrowski@anl.gov; 4 AREVA NP, Université Montpellier 2, Montpellier 34095, France; E-Mail: olivier.lacroix@areva.com

**Keywords:** perovskite, proton conductor, ceramic, autoclave, TGA, IR, Raman, *in situ*

## Abstract

Ceramics, which exhibit high proton conductivity at moderate temperatures, are studied as electrolyte membranes or electrode components of fuel cells, electrolysers or CO_2_ converters. In severe operating conditions (high gas pressure/high temperature), the chemical activity towards potentially reactive atmospheres (water, CO_2_, *etc*.) is enhanced. This can lead to mechanical, chemical, and structural instability of the membranes and premature efficiency loss. Since the lifetime duration of a device determines its economical interest, stability/aging tests are essential. Consequently, we have developed autoclaves equipped with a sapphire window, allowing *in situ* Raman study in the 25–620 °C temperature region under 1–50 bar of water vapor/gas pressure, both with and without the application of an electric field. Taking examples of four widely investigated perovskites (BaZr_0.9_Yb_0.1_O_3−δ_, SrZr_0.9_Yb_0.1_O_3−δ_, BaZr_0.25_In_0.75_O_3−δ_, BaCe_0.5_Zr_0.3_Y_0.16_Zn_0.04_O_3−δ_), we demonstrate the high potential of our unique set-up to discriminate between good/stable and instable electrolytes as well as the ability to detect and monitor *in situ*: (i) the sample surface reaction with surrounding atmospheres and the formation of crystalline or amorphous secondary phases (carbonates, hydroxides, hydrates, *etc*.); and (ii) the structural modifications as a function of operating conditions. The results of these studies allow us to compare quantitatively the chemical stability *versus* water (corrosion rate from ~150 µm/day to less than 0.25 µm/day under 200–500 °C/15–80 bar P_H2O_) and to go further in comprehension of the aging mechanism of the membrane.

## 1. Introduction

The use of hydrogen has been increasing over the last decade. It has been widely considered as a potential energy vector: it can be used in fuel cells to produce electricity or to convert CO_2_ into Syngas or more complex chemicals [1,2,3,4,5,6,7,8,9]. Recently, it has been used to produce better quality fuel and various chemicals from oil. For these applications one needs low-cost, environmentally friendly produced hydrogen. One possible solution is a coupling of the hydrogen production device with a nuclear plant and/or other renewable energy sources such as solar or wind power in order to transform the electricity produced at time where there is no demand (off peak electricity). In this light, high/middle temperature, water electrolysis appears very interesting, especially if the electrolyte and cermet electrodes of the water electrolyser are built of proton conducting membranes. The proton conducting perovskite-like materials, *i.e.*, AB^4+^_1–*x*_Ln^3+^*_x_*O_3−__δ_ (with A: Ba/Sr, B: Zr, Ce and Ln: Y, Yb, In, *etc.*) seem to be a very economical solution: (i) they are reported to operate between 400 and 650 °C, which are the temperature values high enough to avoid expensive noble catalysts but low enough to allow the optimization of the costs of industrial devices (steel selection, working temperature inferior to the H_2_ ignition point); and (ii) they allow the production of dry hydrogen at the cathode site (note, in the case of oxygen-conducting membranes, the hydrogen is obtained at the anode site and is systematically mixed with water) [1,2,3,6,8,9,10,11,12,13,14,15,16,17,18]. 

The efficiency of water electrolysis can be increased if it is carried out under steam conditions [19,20,21]. The combination of high temperature and high water vapor pressure allows an increase in proton conductivity, but can also promote the advancement of reaction with water, and hence the membrane aging. One of the most important criteria to classify a perovskite ceramic as a good gas tight electrolytic membrane is its high mechanical and chemical stability over thousands of hours in severe operating conditions: high temperature, electrical field, gas flow, chemical gradient and vapor pressure cycling. Obviously the higher the chemical gradient/partial pressure, the faster/stronger the diffusion flow [22] and the expected (electro)chemical membrane degradation. Therefore, the structural and chemical stability of a membrane should be determined as a function of operating conditions. Since direct *in situ* measurements are difficult to conduct when a high temperature is combined with gas pressure and flow, most of the necessary sample characterizations are performed *ex situ* far from working conditions, and/or a device is studied after dismounting or *post mortem* [23,24] and references therein. 

In the first part of this article we will present an autoclave platform developed in our laboratory, which allows *ex situ* and *in situ* stability tests to be performed in water steam-electrolysis like conditions. Two autoclaves equipped with sapphire windows allowing *in situ* Raman scattering study are the key elements of this platform. Note that the autoclaves initially allow the incorporation of the protonic species into a host perovskite structure. The complexity of the protonation process, the quantitative and qualitative analyses allowing the differentiation between the bulk and surface proton species as well as the determination of the bulk proton nature, are discussed in our previous work [25]. 

In the second part of this manuscript, the diffraction, Raman, IR, TGA results obtained for four different proton conducting perovskite high dense ceramics: BaZr_0.9_Yb_0.1_O_3−δ_ (BZ:Yb), SrZr_0.9_Yb_0.1_O_3−δ_ (SZ:Yb), BaZr_0.25_In_0.75_O_3−δ_ (BZ:In) and BaCe_0.5_Zr_0.3_Y_0.16_Zn_0.04_O_3−δ_ (BCZ:Y,Zn) will be presented in order to compare their stability aspects. These perovskite compositions are widely studied by many academic and industrial groups up to the demonstrator scale [6,9,12,13,14,15,16,20,21,22,23,24,25,26,27,28,29,30,31,32,33,34,35,36]. They appear promising candidates for various electrochemical applications where a combination of high conductivity and high stability is required. Stability evaluation does not only concern thermodynamic criteria, but also those related to kinetics that depends on the whole object: crystallinity, purity, densification, *etc*. The objective of this manuscript is to quantitatively evaluate the aging rates of high quality ceramics in water steam-electrolysis like conditions using a unique autoclave platform. 

## 2. Results and Discussion

### 2.1. The High Temperature and High Pressure Autoclaves

Table 1 lists the specific working conditions of autoclaves constituting the platform present in Laboratory of Dynamics, Interactions and Reactivity (LADIR). This autoclave platform was developed in direct relation with the study of a water steam electrolyser prototype allowing the low cost of hydrogen production [19,20,21]. The autoclaves were constructed in order to study physical/chemical behavior of potential electrolytes and electrodes in the conditions of reproducing operating/working conditions of such a pressurized electrolyser. 

**Table 1:** Description of the LADIR’s platform of autoclaves.

Autoclave	Volume (L)	Working conditions	Type of study
Ladir	0.27	RT-200 °C, 1–15 bar P_H2O_	protonation/aging test
Celeva (×2)	0.125	RT-315 °C, 1–100 bar P_H2O_	protonation/aging test
Heleva	0.125	RT-600 °C, 1–100 bar P_H2O_	protonation/aging test
*in situ* optic #1	0.05	RT-620 °C, 1–50 bar P_H2O_	protonation/aging tests followed *in situ* by Raman scattering
*in situ* optic #2	0.05	RT-500 °C, 1–30 bar P_H2O_ or P_CO2_, electric field	protonation/aging tests followed *in situ* by Raman scattering with or without electric field

#### 2.1.1. Autoclaves

**Figure 1:**
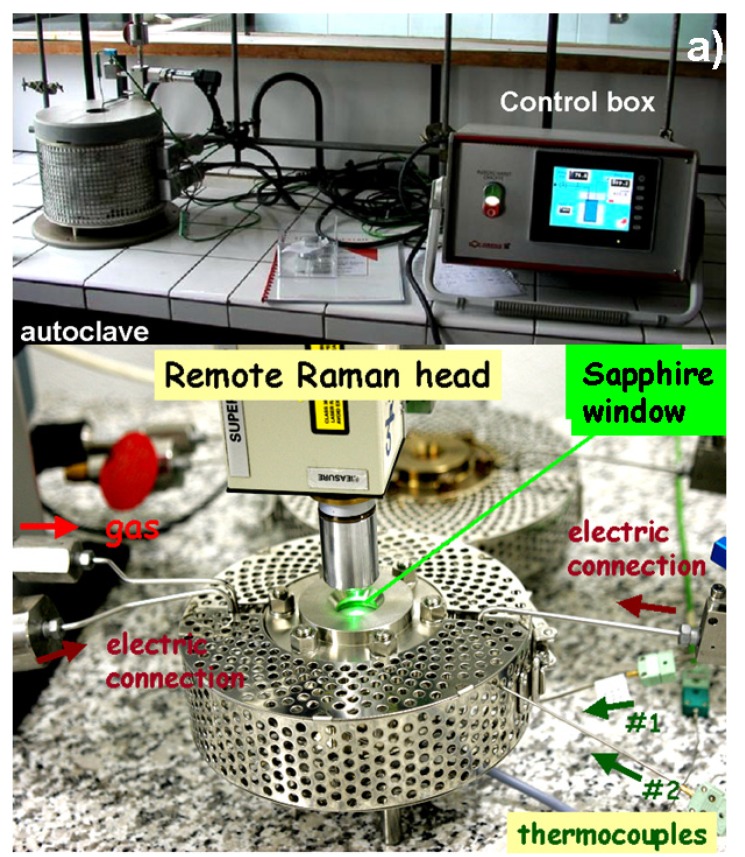
Autoclaves used for high temperature/high pressure protonation and aging: (**a**) Heleva <600 °C, <100 bar P_H2O_ and (**b**) *in situ* optic autoclaves allowing Raman analysis: on the first plan <500 °C, <30 bar of gas pressure; note the gas injection/extraction supply and electric connectors in the autoclave coupled with the remote Raman head equipped with a ×50 Nikon long working distance microscope objective; the autoclave <620 °C, <50 bar of P_H2O_ is shown in the second plan.

As summarized in Table 1, the four non-optic autoclaves are capable of operating between 200 and 600 °C up to 100 bar of water vapor pressure. Each autoclave is equipped with its own electronically controlled heating system (Figure 1). The maximum desired pressure value is achieved by adjusting the temperature and depends on the introduced water quantity. It should be stressed that water under high pressure becomes extremely corrosive above 350 °C. Consequently, the development and use of devices based on P_H2O_ requires experience and know-how for the selection of convenient steel as well as calculation of the admitted stress present under these conditions. 

#### 2.1.2. Raman *in Situ* Autoclaves

The *in situ* Raman autoclaves offer the ability to follow the protonation and aging processes *in real time* by Raman scattering. Raman scattering, largely used on different ordered or disordered perovskites such as ferroelectrics, relaxor ferroelectric or proton/oxygen conductors [37,38,39,40,41,42,43] and references therein, allows the determination of both long and short range order structural modifications. In the case of proton conducting perovskites the host perovskite structure is modified by Ln substitution, that fixes the amount of oxygen vacancies and then by the maximum level of incorporated water/protons [23,37,38,39,40]. The bulk H-content (a few H × 10^−2^ mole/perovskite mole) is largely inferior to the value permitted by the oxygen vacancies. After the protonation process at temperatures below 500 °C, an intense broad band with a maximum of intensity at ~2500 cm^−1^ appears [37]. The origin of such a band is related to the formation of electronic defects due to proton insertion and to the mismatch between the diffusion of interstitial protons and of oxygen atoms/vacancies. Its intensity has been correlated to the protonic species content as well as to the conductivity of a material [37,39]. This band disappears under ~4 GPa hydrostatic pressure [41]. The disappearance of the electronic defects signature is ascribed to the faster homogenisation of oxygen atom/vacancies under high pressure/temperature [40]. Moreover, Raman scattering is also very useful to detect the presence of undesirable covalently bonded secondary phases, even limited to trace amounts and often not detectable by diffraction methods [42]. 

Both of the custom designed and manufactured optic autoclaves presented in Figure 1b, operate as a function of temperature (RT-620 °C for #1 or RT-500 °C for #2) under high pressures: of water vapor or CO_2_, NH_3_, *etc*., and atmosphere. The autoclave chamber can be heated up to 350 and 300 °C, respectively (this fixes the maximum water pressure) and an additional heater allows increasing the sample temperature up to 620 and 500 °C, respectively.

The presence of a sapphire window permits the recording of Raman spectra as a function of operating conditions. The autoclave is coupled with a high sensitivity HE532 (or HE785) Yvon Jobin Raman microspectrometer(s) equipped with a Peltier cooled Synapse (or Andor) CCD detector. The spectrometer is optical fiber coupled to a remote SuperHead^®^ optical head equipped with selected high quality Notch filters and a long working distance microscope objective for sample illumination and light scattering collection. The laser beam is injected from the optical fiber into the optical head. The fix spectral window ranges between 80 and 3300 cm^−1^. The high luminosity and sensitivity of Raman spectrometer as well as the use of a high quality long working distance objective allows the recording of detailed spectra, without significant intensity lost. More technical details are available in [43].

### 2.2. The Study of Chemical and Structural Stability of Proton-Conducting Perovskite Membranes

#### 2.2.1. Characterization of Non-Protonated Ceramics

It should be stressed that the analysis of a ceramic material in its pristine, non-protonated state is crucial in order to detect any small structural and chemical modifications that could appear after autoclave treatment. In order to make such an analysis credible, in our study the multi-approach is based on complementary experimental techniques: X-ray diffraction, TGA, IR and Raman are systematically applied. As we will show, diffraction methods—widely used in the literature to determine the sample purity and quality—are very important but not sufficient on their own.

**Figure 2:**
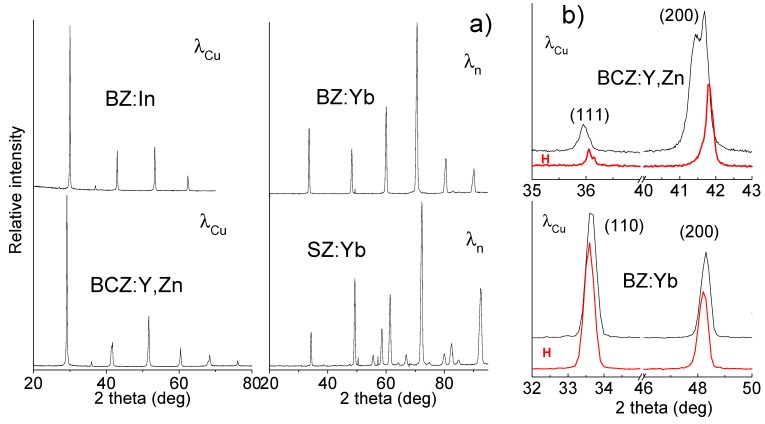
(**a**) The diffraction patterns characteristic of non-protonated BZ:In (X-ray K_α_Cu), BCZ:Y,Zn (X-ray K_α_Cu), BZ:Yb (neutron) and SZ:Yb (neutron); (**b**) Comparison of Bragg peaks characteristic of BCZ:Y,Zn (X-ray K_α_Cu = 0.154 nm) and BZ:Yb (neutron, 0.242 nm) in protonated (H, red) and non-protonated states.

Figure 2a presents the room temperature X-ray or neutron diffraction patterns characteristic of BZ:In, BCZ:Y,Zn as well as BZ:Yb and SZ:Yb, respectively. Each of these four dense ceramics (90% to 98% of theoretical maximum density) shows a pure perovskite phase from a diffraction point of view. The careful analysis of Bragg peak shapes reveal the presence of cubic symmetry with the *Pm3m* space group in the case of BZ:In and BZ:Yb [23,38,39]. The SZ:Yb possesses an orthorhombic symmetry with the Pnma space group. On the contrary, the determination of the symmetry of BCZ:Y,Zn is rather ambiguous. The (111) Bragg peak is not simple (Figure 2b), which clearly shows the non-orthogonal crystallographic system. The literature suggests the rhombohedral symmetry for homologue compounds [44]. However, the evident splitting of (200) reflection rules out this possibility. Two assumptions can be considered: a small monoclinic distortion similar to results presented by Tu *et al*. [44] or a more probable nanomixture of perovskite phases with different degrees of distortions.

**Figure 3:**
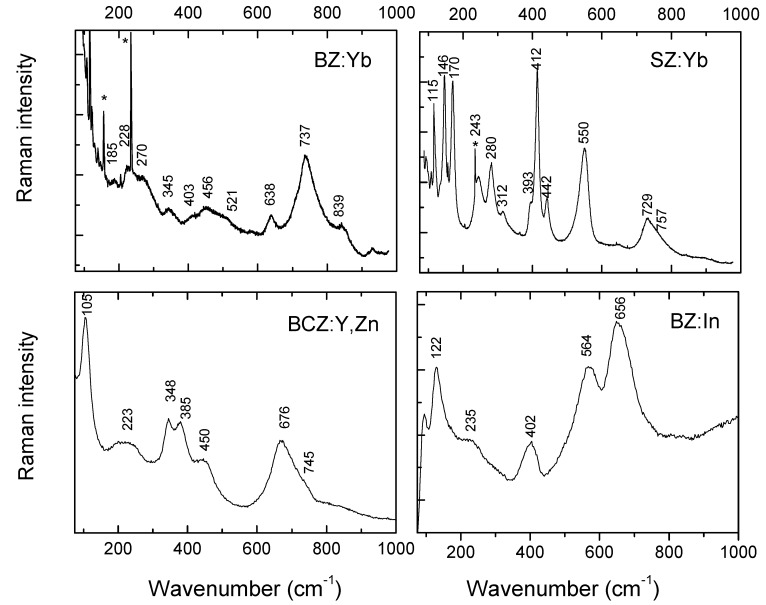
Comparison of Raman spectra characteristic of BZ:Yb, SZ:Yb, BCZ:Y,Zn and BZ:In compounds (see Table 2) in their pristine state before autoclave treatment. The bands at 600–800 and 250–450 cm^−1^ are (more or less coupled) B–O stretching and bending modes, whereas modes below 200 cm^−1^ are lattice modes sensitive to the long range A cation order/symmetry. Asterisks mark the laser plasma lines.

In order to confirm/clarify the diffraction results, and to detect the distortion of the perovskite unit-cell, Raman scattering analysis was performed (Figure 3). Raman spectroscopy is capable of detecting some secondary phases limited to traces or less crystallized phases, in some cases not detectable by common diffraction techniques. Careful characterization of (potential) secondary phases can be found in our previous article [42]. 

The Raman spectrum of a perovskite compound can be considered as characteristic of a (partially) covalently bonded structure [37,41,47]. An ideal ABO_3_ perovskite-like structure (*Pm3m* space group, O_h_) shows no Raman active modes and only three IR bands [37,45,46,47,48]. A low wavenumber range (below ~200 cm^−1^) reveals the vibrations of the A ion network which couple themselves with vibrations modes (T’, R’) of BO_6_ iono-covalent entities leading to the lattice modes. The bending (δ) and stretching (ν) modes of BO_6_ octahedra are expected in the 300–500 cm^−1^ and 600–900 cm^−1^ wavenumber ranges, respectively [45,46,47,48,49,50]. The Raman spectra characteristics of the four perovskite ceramics differ in the number of Raman modes, their position and full width at half maximum (FWHM), which clearly shows different symmetries as well as levels of short-range disorder [47,50]. Consequently, for a given composition, the lower the symmetry, the more Raman modes observed, and the higher their absolute intensity [47,48,49,50,51,52,53]. For instance: 8/7 and 24/28 Raman/IR modes are expected in the case of tetragonal (C_4v_) and orthorhombic (D_2h_) symmetry, respectively [47,46]. 

As can be clearly seen, the Raman spectrum of SZ:Yb, with 12 well resolved modes, confirms the Pnma orthorhombic symmetry determined by neutron diffraction results [38]. The most FWHM values are rather low, especially for the lattice modes involving the A cations motions, which reveals long-range structural order. The broadening of the Zr–O stretching mode located at 730 cm^−1^ is assigned to the octahedron perturbed by oxygen vacancies is an exception. 

The Raman spectrum of BCZ:Y,Zn ceramic is composed of seven rather broad Raman lines. This spectrum cannot be assigned to the monoclinic symmetry [44]—in such a case more Raman lines should be observed—and suggests rather a multiphasic, disordered material. In the case of disordered materials, the existence of nanoregions with distinct local symmetry (and hence various Raman signatures) is usually detected [47] and references therein. The presence of such nanodomains gives rise to the Raman activity depending on the B–O polarizability: for instance the contribution of modes involving Ce– and Zr–O bonds will be much higher than that of Zn–O ones. 

The presence of Raman activity in the case of BZ:In and BZ:Yb can be explained by the local distortions from cubic *Pm3m* symmetry. As mentioned above, an ideal perovskite-like structure shows no Raman active modes [37,45]. This reveals the existence of a more or less disordered cubic symmetry—mean matrix—in which the nanoregions of lower local symmetry are embedded. This is interesting in the case of BZ:Yb which does not differ much from pure BaZrO_3_ and we do not detect any modes in the lattice mode region. The highly ionic Ba-based sublattice interacting in the long range seems to respect the cubic symmetry. The structural disorder is then limited to the covalent octahedron subblatice. In the case of BZ:In and BCZ:Y,Zn with a higher level of substitutions (Table 2), the disorder concerns both ionic and covalent sublattices and the well-defined ~110 and 120 cm^−1^ modes (Figure 3) exclude the cubic symmetry. 

**Table 2:** Characterization of different proton conducting electrolytic membranes after high temperature/high pressure autoclave treatment.

Compound	BCZ:Y,Zn	BZ:In	BZ:Yb	SZ:Yb
Composition	BaCe_0.5_Zr_0.3_Y_0.16_Zn_0.04_O_3−δ_	BaZr_0.25_In_0.75_O_3−δ_	BaZr_0.9_Yb_0.1_O_3−δ_	SrZr_0.9_Yb_0.1_O_3−δ_
State before treatment	High density (97%) ceramic pellet, middle brown	Dense (90%) ceramic pellet, light brown	High density (97%) ceramic pellet, middle brown	High density (98%) ceramic pellet, middle brown
Autoclave Treatment	500 °C/10 bar H_2_O/30 h	300 °C/80 bar H_2_O/5 days	200 °C/15 bar H_2_O/23 days	200 °C/15 bar H_2_O/5–23 days OR *500* *°C/80 bar H_2_O/5 days*
Habit	Surface slightly crumbled	Total ceramic crumbling	Surface slightly crumbled	Thin surface layer OR *no change*
Structural/chemical changes	Coexistence of perovskite phase and BaCO_3_, CeO_2_, Ba(OH)_2_·H_2_O →Partial decomposition	BaCO_3_, ZrO_2_, Ba(OH)_2_·H_2_O, In_2_O_3_ →Total decomposition	Coexistence of perovskite phase and traces of BaCO_3_, Ba(OH)_2_·H_2_O →Partial decomposition of surface—Bulk OK	Traces of SrCO_3_, and Sr(OH)_2_·H_2_O on the surface
Symmetry (non –H)	Distorted rhombohedral & multiphased	Distorted cubic	Cubic	Orthorhombic
Symmetry (H)	Cubic	Decomposition	Cubic	Orthorhombic
Decomposition rate	~30 µm/day	>150 µm/day	~10 µm/day	<0.25 µm/day

The Raman spectra observed in Figure 3 seem to confirm a perovskite phase as determined by diffraction results, without the presence of any secondary phases such as carbonates and/or hydroxides. However, they do reveal nano-heterogeneity and short-range disorder.

**Figure 4:**
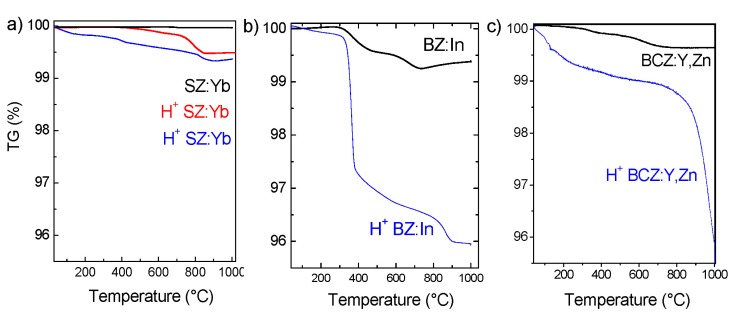
TGA results characteristic of (**a**) SZ:Yb ceramics before autoclave treatment (black upper curve) and after protonation at 500 °C/80 bar H_2_O (red middle curve) and 200 °C/15 bar H_2_O (blue bottom curve); (**b**) BZ:In ceramics before (black upper curve) and after (blue) autoclave treatment at 300 °C/80 bar H_2_O; (**c**) BCZ:Y,Zn before and after 30 h at 500 °C/10 bar H_2_O. In order to simplify the comparison the TGA data are presented in the same scale.

Finally, to complete the analysis of non-protonated ceramics, thermogravimetric (TGA) curves (Figure 4) and IR spectra (see further) were recorded. As seen in Figure 4a, the pristine SZ:Yb does not show any mass loss during the applied thermal treatment, which clearly shows that this sample is composed of the single, chemically stable perovskite phase. Similar behavior is detected for BZ:Yb. On the contrary, BZ:In (Figure 4b) and BCZ:Y,Zn (Figure 4c) ceramics heated to a temperature of 1000 °C show an unexpected mass loss above 300 °C, which reveals the presence of some secondary phases, mostly Ba(OH)_2_·*n*H_2_O [42,54]. As it can be clearly seen, the content of secondary phases is the highest in BZ:In ceramics, in good agreement with IR results. 

#### 2.2.2. Protonated Ceramics

Table 2 summarizes the results of the characterization of the four proton conducting perovskite membranes tested using the autoclave platform. 

**Figure 5:**
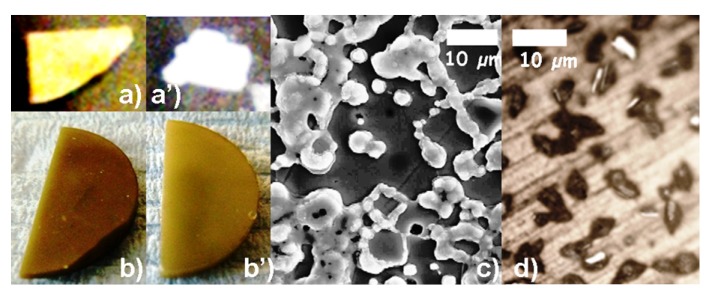
Comparison of ceramic membranes before and after autoclave treatment, “naked eye” analysis shows decomposition of BZ:In pellet (**a**) (diameter ~13 mm) into a powder (**a’**); BCZ:Y,Zn ceramic (**b**) (diameter ~12 mm) keeps the mechanical properties, a color change is detected (**b’**). The microscope analysis reveals the surface phase formation on BCZ:Y,Zn (SEM) (**c**) and SZ:Yb (optical) (**d**) ceramic grain boundary.

After the autoclave treatment, a simple eye examination of the ceramic membranes provides a first estimation of stability. In the extreme cases—see Figure 5a,a’— the dense ceramic is completely crumbled or even powdered. Such a total loss of mechanical properties was detected for BZ:In ceramic after 300 °C/80 bar P_H2O_/5 days treatment. More often, the visual and mechanical aspects of the ceramics are very similar to that observed before thermal/pressure treatment. As presented in Figure 5b,b’ sometimes a subtle color change can be detected, for example in the case of BCZ:Y,Zn according to the modification of electronic defects. Consequently, the careful analysis of treated ceramics is essential prior to any further study such as conductivity measurements for example. As we previously discussed [23], the systematic lack of quantitative and qualitative study of hydrated samples give rise to important misunderstanding of fundamental aspects of proton conducting materials. 

Careful analysis using an optical or scanning electron microscope allows the detection of possible secondary phases formed on the ceramic surface in the case of BCZ:Y,Zn (Figure 5c) and SZ:Yb protonated at 200 °C under 15 bar (Figure 5d). These phases appear to form preferentially at the grain boundary regions. A higher reactivity of the grain boundary region can easily be understood. The high temperature required to sinter pure zirconate can be lowered by adding elements with lower oxide melting temperatures (e.g., replacement Zr/Zn) [55] or by keeping a small excess of Ba/Sr in order to promote a high grain surface diffusion. Incomplete elimination of this (small) excess (0 to 0.3% of the Ba/Sr content, typically) can lead to the high reactivity with water. Segregation at the grain boundary of the substituted atoms also should increase the reactivity. Raman, IR, EDX and TG analyses or likely Transmission Electronic Microscopy are necessary to identify these secondary phases as well as to verify if they are limited to surface traces or are also present into a ceramic bulk. 

Figure 2b shows an example of diffraction data characteristic of protonated and non-protonated ceramics. As it can be clearly seen, important differences are detected in the case of BCZ:Y,Zn ceramic. Namely, after the protonation, the Bragg peak splitting is no longer observed which reveals the structural transformation into cubic symmetry as previously demonstrated for series of perovskites [37]. This shows that the insertion of protonic species leads to the symmetrization of crystal structure. Note that TGA and neutron scattering results reveal very low (less than H × 10^−2^ mole/perovskite mole typically) content of bulk protons in perovskite ceramics [3,24,25,38,39,40]. The neutron diffraction patterns characteristic of protonated and non-protonated BZ:Yb (Figure 2b) and SZ:Yb are almost the same. Only small shift of Bragg peak positions can be detected which shows that only the unit-cell parameters (and volume) are slightly changed [38,39]. Such small modifications of crystal structure have been confirmed by Raman analysis [38]. 

TGA analysis is a very useful technique to determine the content and type of protonic species incorporated by a ceramic during the operation [3,24]. Figure 4 compares the TGA curves recorded for SZ:Yb (a), BZ:In (b) and BCZ:Y,Zn (c) dense ceramics. As it can be clearly seen, the type of incorporated protonic species in the case of SZ:Yb ceramic strongly depends on operating conditions. The treatment at 200 °C under 15 bars of H_2_O, favors the presence of surface moieties and hydroxylation of the sample (and carbonation if most of the residual H_2_CO_3_ has not been eliminated from water) (see Reference [56] for more details). On the contrary, the use of high pressure: 80 bar and high temperature, *i.e.*, 500 °C, allows the successful incorporation of bulk protons without a significant presence of secondary phases. In the case of BZ:In compounds, an important enhancement of Ba(OH)_2_·*n*H_2_O phases is detected. As mentioned above, the pristine, non-protonated ceramic already contains surface secondary phases. Once the ceramic is exposed to severe operating conditions (300 °C and 80 bar of water vapor pressure during 5 days), the total loss of mechanical properties is observed (Figure 5a’), and the sample is decomposed structurally and mechanically. The BCZ:Y,Zn ceramic shows an almost continuous mass loss with the temperature increase revealing successive departure of water (>200 °C), hydroxides (>400 °C), bulk proton (~600 °C) and decomposition of carbonates above 800 °C.

The TGA and IR/Raman data show that during treatment in severe operating conditions two competing processes take place: bulk protonation and hydroxylation. The extension of the second mechanism leads to a decomposition of the ceramic starting from the surface due to the formation of secondary phases such as hydroxides and/or carbonates. As discussed in detail in [25], the initial sample state (as well as the operating conditions) plays a key role in this competition. The lack of secondary phases and high density of a ceramic is crucial to avoid hydroxylation. 

**Figure 6:**
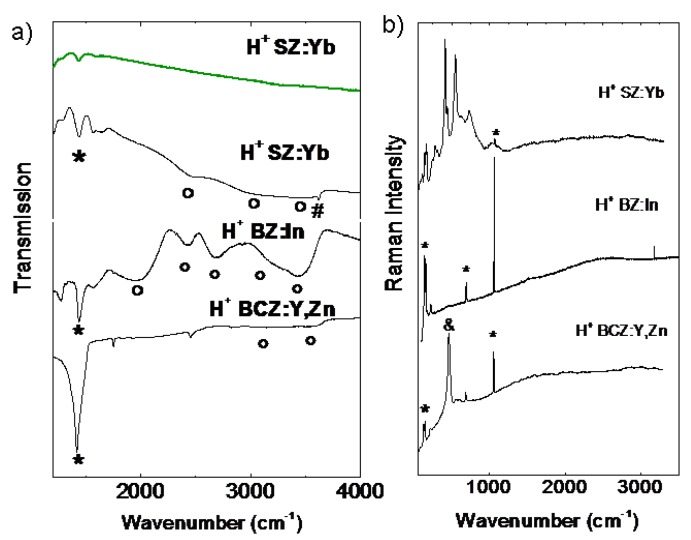
(**a**) Representative IR signatures of ceramics after autoclave treatment. The phases formed by reaction with water under high pressure are detected: * carbonates; ° hydrogen-bonded protonic species; ^#^ OH– isolated vibrator. Protonated SZ:Yb spectra have been recorded in transmission through polished ~150 µm thick membrane; protonated BZ:In and BCZ:Y,Zn ceramics have been ground and dispersed in CsI pellets for transmission measurements. Note the spectrum of SZ:Yb protonated at 500 °C/80 bar (green) is free from secondary phases and shows no signature in OH stretching region. (**b**) Raman signature of ceramics after autoclave treatment: SZ:Yb (200 °C/15 bar), BZ:In (300 °C/80 bar) and BCZ:Y,Zn (500 °C/10 bar). The presence of carbonates * and CeO_2_ & is clearly visible.

The TGA results are in very good agreement with the IR transmission results presented in Figure 6a. The SZ:Yb ceramic, protonated at high temperature and high pressure (green spectrum), is free from carbonates and hydrogen-bonding species. The lack of any signature in the OH-stretching region can be explained by the presence of bulk interstitial protons with ionic, free of covalent bond nature [25]. On the contrary, the IR spectrum of the same sample protonated at conditions favoring hydroxylation reveals clear SrCO_3_ traces and an important spectroscopic signature in the OH stretching region: OH^−^ (3600 cm^−1^), (OH–) H_2_O (3500 cm^−1^), physisorbed H_2_O (3200 cm^−1^) and ABC bands (1800 cm^−1^, 2400 cm^−1^ and 3000 cm^−1^) characteristic of strongly H-bonded species [42,56].

In the case of the protonated powdered BZ:In sample, the IR spectrum, in perfect agreement with the Raman one [54], points to the total decomposition of the perovskite structure. The complex IR spectrum clearly shows different H-bonding species: hydrates, hydroxides and carbonates [42]. The Raman spectrum reveals the presence of secondary phases, mostly BaCO_3_, rarely LnCO_3_. The IR spectrum of BCZ:Y,Zn ceramic shows an important signature of BaCO_3_ after 30 h treatment at 500 °C under 10 bar of H_2_O and only traces of hydroxides/hydrates in the high wavenumber range. 

#### 2.2.3. *In Situ* Monitoring of Protonation/Operating—Stability Test

**Figure 7:**
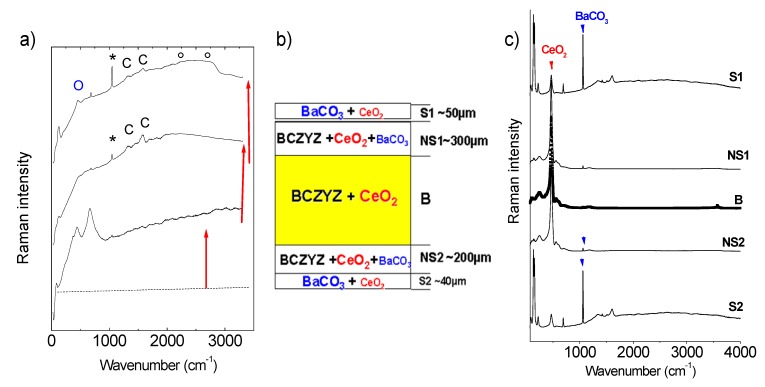
(**a**) *in situ* Raman spectra recorded for BCZ:Y,Zn ceramic at 500 °C/10 bar H_2_O as a function of time (0, 5 and 30 h) with the progressive formation of the Fluorescence background arising from electronic defect(s) associated to inserted protons (Raman profilometry). Note, the formation of O CeO_2_,* BaCO_3_ and ° H-bonded secondary phases; (**b**,**c**) Profilometry Raman analysis of a BCZ:Y,Zn (studied in **a**) ceramic fresh cross-section and broken after 30 h treatment.

The protonation process, performed under steam water electrolyser conditions, can be successfully followed *in situ* by Raman scattering, using our optic autoclave device. We will mainly discuss the case of the BCZ:Y,Zn sample. Its medium position in the stability range scale—between the totally decomposed BZ:In and very stable SZ:Yb—makes it the most characteristic example. Figure 7a shows the Raman spectra recorded at 500 °C/10 bar as a function of operating time. 

The first spectrum recorded after 2 h (Figure 7a) is typical of a protonated perovskite material. Namely, the absolute Raman intensity decreased and the broad background is observed [26]. Over time, the intensity of Raman modes characteristic of perovskite framework decreases systematically; simultaneously the signature of secondary phases increases. Consequently, after 30 h of operating the surface, the strong signature of BaCO_3_ and CeO_2_, dominates Raman spectrum. Note, the Raman intensity is not directly proportional to phase content: in the case of BCZ:Y,Zn, the detected ceria secondary phase is more Raman active than the perovskite structure containing the protons. It is clear that after 30 h, the surface of BCZ:Y,Zn ceramic is a complex mixture of perovskite phase and secondary phases. 

The important question is: if these undesirable modifications are only limited to the surface, do they progressively corrode the ceramic bulk. In order to clarify this point we have performed the Raman profilometry on the fresh cross-section fracture of protonated BCZ:Y,Zn ceramic (Figure 7b,c). The obtained results are summarized in Figure 7b. The secondary phases are detected throughout the ceramic section. It is possible to distinguish some characteristic regions: (i) surface that mostly contains BaCO_3_ as a second phase (Figure 7c); (ii) the near-surface that contains mostly CeO_2_ and traces of BaCO_3_; and (iii) ceramic bulk rich in CeO_2_. It should be stressed that for both surface regions (S1 and S2) an important loss of mechanical properties was detected and observed as a crumbling of the sample upon contact. Since these secondary phases accompany the perovskite phase, the partial decomposition of the sample is observed. 

### 2.3. Corrosion Rate and Mechanisms

The profilometry results allowed us to estimate the decomposition rate of the ceramic. In this estimation, the powder-like external layer, the most decomposed, will be considered only. Consequently, the decomposition rate characteristic at 500 °C/10 bar H_2_O of BCZ:Y,Zn is approximately 30 µm/day. Similar estimation gives 10 µm/day in the case of BZ:Yb and 0.25 µm/day for SZ:Yb. In the case of BZ:In—totally decomposed after five days at 300 °C/80 bar H_2_O—the rate is very important, approximately 150 µm per day. 

An important challenge is to understand the main reason of the material’s instability. The work presented seems to suggest, first of all, that the choice of Alkali Earth element is important: the Sr-based membrane is more structurally, mechanically, and chemically stable than the Ba-based ones. Secondly, another stability aspect is related to the B-substitution level. Our results suggest that the lower the substitution level, the higher the membrane stability. Note the aim of high substitution level in the case of BZ:In was to significantly increase the amount of oxygen vacancies and then the bulk proton content. However, it appears that that an increased level of substitution leads to premature aging. In addition, the type of substituting elements: Yb or Ce/Zn/Y seem to play an important role. The cerium is well known to be instable *versus* water and CO_2_ atmosphere and both Y and Zn elements easily form hydroxides. Further study is necessary to clarify the aspects accelerating the aging process of the material, for example, the careful investigation of a system with a slightly different substitution level. 

**Figure 8:**
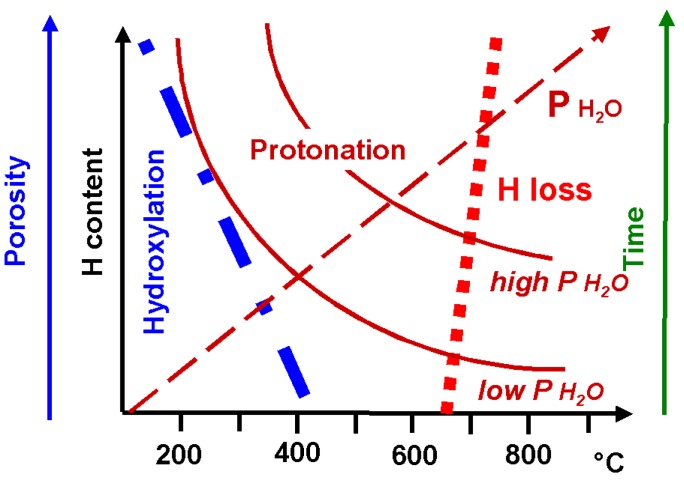
Hydroxylation *vs*. Protonation: high porosity of ceramic and temperature below 400 °C enhance hydroxylation; on the contrary high ceramic density, high temperature and high water pressure promote proton incorporation; the proton loss takes place over 650 °C.

To summarize, a high interaction of alkali earth elements with water and CO_2_ is in the apparent origin of the aging mechanism, but the corrosion rate depends upon structure and Zr substitution. Very easy formation of alkali earth–based secondary phases makes the perovskite structure more fragile. Because of the loss of A elements, the perovskite structure transforms into the ZrO_2_ and/or CeO_2_ oxides. Our previous results clearly show that for a given composition [56] only highly dense samples, free from any traces of secondary phases in a pristine state, are stable in severe operating conditions for many weeks. Note, that as we state many times, the choice of operating conditions is crucial in order to decrease, as much as possible, the hydroxylation tendency. Figure 8 summarizes the sample/operating conditions requirements in order to enhance the bulk protonation. 

**Figure 9:**
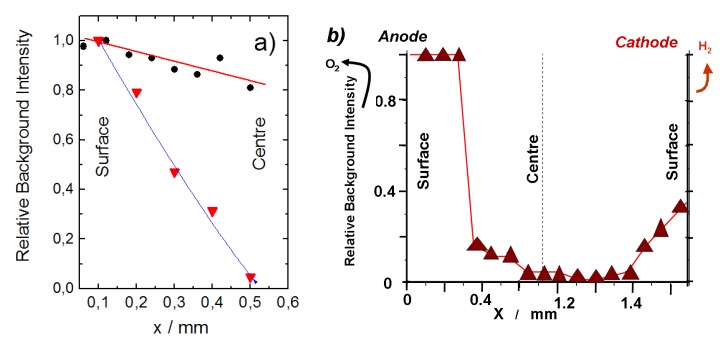
(**a**) Progressive formation of the Fluorescence background arising from electronic defects associated to inserted protons (Raman profilometry) measured on SZ:Yb (red curve) and BZ:Yb (blue curve) ceramic membrane half section protonated in autoclave, respectively for short (protonation level decreases from the surface to the center according to Fick’ laws) and long duration (homogeneous protonation); (**b**) Raman background profile measured along the section of a ceramic membrane (SZ:Yb electrolyte) used for water electrolysis and H_2_ production at 550 °C for tenths of hour [56]. Pt grids have been used anode (water supply side) and cathode (H_2_ production side). Note the high intensity on the water-rich side.

### 2.4. Towards Analysis of Electrochemical Dispositive

The examples discussed above concern the electrolyte only with both sides exposed to the same atmosphere. In an electrolyser or a fuel cell the conditions are in fact more severe: both electric field and a water gradient are present. Figure 9 compares the Raman profilometry results measured for “simple” SZ:Yb and BZ:Yb electrolyte membranes after their exposure in autoclaves for various times as well as *post mortem* for a dispositive using SZ:Yb as an electrolyte used in the water steam electrolyser at 600 °C. This comparison clearly shows important differences. The most significant change is related to the asymmetry of the background intensity associated to electronic defect-proton distribution measured along the electrolyser membrane section. The presence of the cathode and anode where important oxidation/reduction reactions take place makes the stability equilibrium more complex. As expected, the material close to the anode side where a water pressure is provided shows a saturation of the background signal. This signal is minimal at the center of the membrane and increases on the cathode side. Note, for a ceramic exposed to high water pressure in the 500–600 °C region, the background intensity is low. Obviously the gradients experienced by the ceramic membrane play an important role on the local structure and (electronic) defect formation. 

The next step of our research: determination of the aging mechanism of real components and it appears as a highly interesting challenge. 

## 3. Experimental Section

Highly dense Yb-modified SrZrO_3−δ_ and BarZrO_3−δ_ ceramic pellets were produced by chemical route and sintered at 1550 °C [19,20,56,57]. The BaZr_0.25_In_0.75_O_3−__δ_ ceramics were prepared by solid-state reaction and sintered at 1420 °C [54]. The BCZ:Y,Zn ceramics were prepared also using the solid state reaction method based on that described by Tao and Irvine [55,58]. 

The incorporation of protons into the host perovskite structure as well as stability tests were performed using high temperature and high water vapor pressure autoclaves (see Section 2.1.1). In order to minimize, as much as possible, the contribution of CO_2_/CO_3_^2−^ the used high purity water was previously decarbonated. Moreover, the autoclave tightness makes any contact with external atmosphere impossible. 

X-ray diffraction measurements were performed using Rigaku D-Max Powder Diffractometer with CuKα radiation. The neutron diffraction patterns were recorded for SZ:Yb and BZ:Yb ceramics using the Cold Neutron Two-Axis Diffractometer PYRRHIAS G 4-1 (LLB, Orphee French Neutron Facility). 

TG analysis was performed using a Setaram Setsys instrument in the 25–1000 °C temperature range, with a heating rate of 5 °C/min. A Pt crucible and He atmosphere are used to maximize the heat transfer and hence, the measurement accuracy.

Raman spectra prior to the Raman profilometry were recorded using HE532 (80–3200 cm^−1^ spectral range) and/or HR 800 Raman (5–>4000 cm^−1^ spectral range) microscpectrometer Horiba Jobin Yvon Raman microspectrometers using 458 nm, 514 nm and 532 nm excitation laser lines. 

IR transmission spectra were recorded using FT Equinox 55 Irscope Bruker Optics microspectrometer. Powder dispersed in CsI matrix and/or polished thin ceramics are used.

More experimental details are available in our previous works [19,20,37,56].

## 4. Conclusions

The understanding of the efficiency loss mechanism is a key point in optimizing the use of proton-conducting perovskites as electrolytic membranes as part of the Hydrogen Economy. Consequently, we have performed accelerating stability tests using a unique, custom designed and made autoclave platform, which allows measurements as a function of high temperature (up to 620 °C) and under high water vapor (up to 100 bar)/gas (up to 30 bar) pressure. Coupling the autoclave with a Raman micro-spectrometer allows *in situ* Raman study. The comprehensive Raman results are compared with those of X-ray or neutron diffraction. In addition, IR and TG analysis have also been performed on four proton conducting perovskite ceramics considered as the most promising candidates for electrochemical applications: BaZr_0.9_Yb_0.1_O_3−δ_ (BZ:Yb), SrZr_0.9_Yb_0.1_O_3−δ_ (SZ:Yb), BaZr_0.25_In_0.75_O_3−δ_ (BZ:In) and BaCe_0.5_Zr_0.3_Y_0.16_Zn_0.04_O_3−δ_ (BCZ:Y,Zn), allowing us to compare their mechanical, structural and chemical stabilities. These techniques make the detection and identification of different secondary phases possible: carbonates, hydroxides, *etc*., even if they are limited to the traces, not normally detectable by diffraction methods. Due to the Raman profilometry, it is possible to determine if any structural/chemical modifications are limited to the surface or are present in the bulk as well. 

Premature aging is observed for Ba-based samples with a high substitution level of the Zr-site. The highly dense SZ:Yb ceramic with low substitution level and low local distortion (see FWHM) appears more stable. Note, the use of this material as an electrolyte allows the successful hydrogen production in a water steam electrolyser prototype. The structure with the highest level of oxygen vacancies and the lowest melting temperature (In) is less stable. Our results also show that the operating conditions: temperature, pressure, and duration play a very important role in membrane stability. Protonation and proton conduction processes are in strict competition with hydroxylation. Hydroxylation—favorites by traces of secondary phases in pristine material and low-density—high active surface area, significantly enhances the membrane instability. 

The first tests performed on real electrochemical devices, tested in water steam electrolyser conditions, under an electric field gradient, reveal additional complex processes which make the understanding of the aging mechanism more difficult. However, this challenging study has to be undertaken if we want to use low cost, environmentally friendly-produced Hydrogen as a main energy vector in the future. 
